# G-protein coupled estrogen receptor (GPER1) activation promotes synaptic insertion of AMPA receptors and induction of chemical LTP at hippocampal temporoammonic-CA1 synapses

**DOI:** 10.1186/s13041-023-01003-3

**Published:** 2023-01-28

**Authors:** Leigh Clements, Amy Alexander, Kirsty Hamilton, Andrew Irving, Jenni Harvey

**Affiliations:** 1 Division of Systems Medicine, University of Dundee, Ninewells Hospital and Medical School, Dundee, DD1 9SY UK; 2 Faculty of Arts, Science and Technology, The University of Northampton, Northampton, NN1 5PH UK

**Keywords:** Estrogen, GPER1, Synaptic transmission, AMPA receptor, Synaptic plasticity, Temporoammonic

## Abstract

It is well documented that 17β estradiol (E2) regulates excitatory synaptic transmission at hippocampal Shaffer-collateral (SC)-CA1 synapses, via activation of the classical estrogen receptors (ERα and ERβ). Hippocampal CA1 pyramidal neurons are also innervated by the temporoammonic (TA) pathway, and excitatory TA-CA1 synapses are reported to be regulated by E2. Recent studies suggest a role for the novel G-protein coupled estrogen receptor (GPER1) at SC-CA1 synapses, however, the role of GPER1 in mediating the effects of E2 at juvenile TA-CA1 synapses is unclear. Here we demonstrate that the GPER1 agonist, G1 induces a persistent, concentration-dependent (1–10 nM) increase in excitatory synaptic transmission at TA-CA1 synapses and this effect is blocked by selective GPER1 antagonists. The ability of GPER1 to induce this novel form of chemical long-term potentiation (cLTP) was prevented following blockade of *N*-methyl-d-aspartate (NMDA) receptors, and it was not accompanied by any change in paired pulse facilitation ratio (PPR). GPER1-induced cLTP involved activation of ERK but was independent of phosphoinositide 3-kinase (PI3K) signalling. Prior treatment with philanthotoxin prevented the effects of G1, indicating that synaptic insertion of GluA2-lacking α-amino-3-hydroxy-5-methyl-4-isoxazolepropionic acid (AMPA) receptors underlies GPER1-induced cLTP. Furthermore, activity-dependent LTP occluded G1‐induced cLTP and vice versa, indicating that these processes have overlapping expression mechanisms. Activity‐dependent LTP was blocked by the GPER1 antagonist, G15, suggesting that GPER1 plays a role in NMDA‐dependent LTP at juvenile TA‐CA1 synapses. These findings add a new dimension to our understanding of GPER1 in modulating neuronal plasticity with relevance to age-related neurodegenerative conditions.

## Introduction

Increasing evidence indicates that estrogen has cognitive enhancing effects as lack of estrogen is associated with cognitive impairments, whereas administration of estrogen enhances learning and memory [10, 64]. The most common estrogen is 17β estradiol (E2), and the biological actions of E2 are mainly attributed to activation of two classical estrogen receptors (ER), ERα and ERβ. These ERs are steroid receptors that mediate the genomic actions of estrogens by functioning as transcription factors. Increasing evidence indicates that rapid non-genomic effects of estrogens are mediated by receptors expressed at the plasma membrane. Recent studies have identified the G-protein coupled estrogen receptor 1 (GPER1) is expressed at the plasma membrane [23, 25] and it is likely to contribute to the non-genomic actions of E2 [3].

Several studies have evaluated the neuronal distribution of GPER1 and high levels of GPER1 expression have been identified in cortical and hippocampal brain regions [4, 6, 14]. Ultrastructural analysis has observed GPER1 expression at hippocampal dendritic spines and postsynaptic density [1, 67], which has fuelled the possibility that GPER1 regulates excitatory synaptic function. Like other hormones, estrogens markedly influence the function of hippocampal synapses, as exposure to E2 facilitates NMDA responses and the magnitude of long-term potentiation (LTP) at Schaffer-collateral (SC)-CA1 synapses [24, 35, 59]. Accumulating evidence suggests that GPER1 contributes to the rapid synaptic actions of E2, as the selective GPER1 agonist, G1 induces a persistent increase in excitatory synaptic transmission at SC-CA1 synapses [58]. GPER1 activation also induces a novel form of hippocampal long-term depression (LTD), via a process that requires co-activation of mGluRs and internalisation of AMPA receptors at SC-CA1 synapses [15].

It is known that in addition to the SC input, hippocampal CA1 pyramidal neurons are directly innervated by the temporoammonic (TA) path that extends from the entorhinal cortex (layer III), with synaptic connections formed on distal dendritic regions of CA1 neurons. The TA input to CA1 neurons is hypothesised to have discrete roles in hippocampus-dependent learning and memory relative to the classical SC input [16]. Recent in vitro brain slice studies have demonstrated that TA-CA1 and SC-CA1 synapses are also differentially regulated by various neuromodulators including the hormones leptin and estrogens [19, 41, 45]. Indeed, E2 is capable of inducing LTP at juvenile TA-CA1 synapses via a process involving activation of ERα [19]. Emerging evidence indicates that GPER1 modulates excitatory synaptic transmission at SC-CA1 synapses [15, 38, 48, 58, 67], however the contribution of GPER1 to the effects of E2 at hippocampal TA-CA1 synapses is unclear.

Here we have explored the role of GPER1 in modulating juvenile TA-CA1 synapses and provide the first compelling evidence that low nM concentrations of the selective GPER1 agonist G1 can induce a novel form of cLTP at this synapse. In contrast, G1 had no effect on excitatory synaptic transmission at juvenile SC-CA1 synapses. The effects of G1 are due to GPER1 activation as its effects are blocked by the GPER1 antagonists G15, and G36. GPER1-induced cLTP also requires NMDA receptor activation and involves a postsynaptic mechanism of expression. Moreover, an extracellular signal-regulated kinase (ERK)-dependent pathway plays a key role, as selective inhibitors of ERK, but not phosphoinositide 3-kinase (PI 3K) signalling, blocked the effects of G1. The ability of G1 to increase synaptic efficacy required the insertion of GluA2-lacking AMPA receptors, as selective blockade of these receptors with philanthotoxin prevented G1-induced cLTP in hippocampal slices. In parallel studies, G1 increased the synaptic density of GluA1-containing AMPA receptors in cultured hippocampal neurons. GPER1-induced cLTP exhibits similar expression mechanisms to activity-dependent LTP as G1-induced cLTP occluded HFS-induced LTP and vice versa. Moreover, GPER1 activation is involved in NMDA-dependent LTP at TA-CA1 synapses as HFS-induced LTP was blocked by the GPER1 antagonist, G15. Together these data indicate that activation of GPER1 induces a novel form of cLTP at juvenile TA-CA1 synapses.

## Materials and methods

### Statement of ethics

All experimental work complied with the UK Animals (Scientific Procedures) Act 1986 and was approved by the University of Dundee Animal Welfare and Ethical Review Committee. Experiments were performed on tissue from male Sprague Dawley rats. Rats had access to food and water ad libitum and were kept in a 12 h daily light–dark cycle. Care was taken to minimize the number of animals used to the minimum.

### Hippocampal slice preparation and electrophysiology

Hippocampal slices (350 μm) were prepared from juvenile (11–22 days old) male Sprague–Dawley rats, as before [19, 46]. Briefly, animals were killed by schedule 1 procedures in accordance with the UK (Scientific Procedures Act, 1986) legislation. Brains were rapidly removed and placed in ice-cold artificial cerebrospinal fluid (aCSF) containing (in mM): 124 NaCl, 3 KCl, 26 NaHCO_3_, 1.25 NaH_2_PO_4_, 2 CaCl_2_, 1 MgSO_4_, and 10 d-glucose and bubbled with 95% O_2_ and 5% CO_2_. Once prepared, parasagittal hippocampal slices were left to recover at room temperature in oxygenated aCSF for 1 h before use.

As TA-CA1 synapses are electrotonically remote from the CA1 cell somata, standard extracellular recordings of local field excitatory postsynaptic potentials (fEPSPs) were used to monitor synaptic transmission at TA-CA1 synapses [46]. In brief, recording pipettes containing aCSF (∼3–5 MΩ) were placed in the stratum lacunosum-moleculare region to record TA-CA1 responses [49]. The CA3 region was removed to prevent indirect activation of the tri-synaptic pathway, and dopamine (100 μM) was routinely applied for 5 min at the end of experiments, to pharmacologically verify stimulation of the TA input [41]. The direct TA pathway was stimulated at 0.033 Hz using a stimulus intensity that evoked peak amplitude ∼50% of the maximum response. For SC-CA1 recordings, the recording pipette was placed in the CA1 region, and responses were evoked by stimulation of Schaffer-collateral fibres. Synaptic field potentials were low-pass filtered at 2 kHz and digitally sampled at 10 kHz. A high frequency stimulation (HFS; 100 Hz for 1 s) protocol was used to induce LTP. For paired pulse facilitation studies, two consecutive stimuli with an inter-stimulus interval of 50 ms were delivered and the paired pulse ratio (PPR) was calculated as the ratio of the slope of the second EPSP relative to the first EPSP.

The slope of the evoked fEPSPs was measured and expressed relative to the preconditioning baseline with 4 consecutive fEPSP slope measurements averaged. Data were monitored online and analyzed offline using the WinLTP program [8]. The degree of G1 or stimulus effect was calculated before addition of dopamine and expressed as a percentage relative to baseline ± S.E.M. For antagonist experiments, all slices were preincubated with pharmacological inhibitors for at least 15 min before agonist application or stimulus induction. In control experiments, all inhibitors were applied for a minimum of 60 min to examine the effect on basal synaptic transmission. Statistical analyses were performed by comparing the 5 min baseline period before the addition of G1 or stimulus induction protocol or application of pharmacological inhibitor with the 5 min period before dopamine application using repeated measures analysis of variance. Data was subsequently compared with interleaved control slices using a paired *t*-test. The degree of cLTP was calculated 35–40 min after delivery of HFS and expressed as a percentage of baseline ± SEM. In PPR studies, mean ratios were compared and statistical analysis was conducted using a paired *t*-test (2-tailed; 95% confidence interval). *p* < 0.05 was considered significant with *n* representing the number of slices used from different animals.

### Hippocampal cell culture

Hippocampal cultures were prepared as previously [45]. Briefly, neonatal Sprague–Dawley rats (P1-3) were killed by cervical dislocation in accordance with Schedule 1 of UK Animals (Scientific Procedures Act, 1986). Hippocampi were removed, and after washing in HEPES-buffered saline comprising (in mM) 135 NaCl, 5 KCl, 1 CaCl_2_, 1 MgCl_2_, 10 HEPES, and 25 D-glucose (pH 7.4), the hippocampi were treated with papain (1.5 mg ml^−1^; Sigma Aldrich, UK) for 20 min at 37 °C. Dissociated cells were plated onto sterile dishes (35 mm diameter; Greiner Bio-One Ltd., UK) treated with poly-d-lysine (20 μg ml^−1^; 1–2 h). Cultures were maintained in serum replacement medium (B27; Thermo Fisher, UK) in a humidified atmosphere of 95% O_2_ and 5% CO_2_ at 37 °C for up to 2 weeks.

### Immunocytochemistry

Immunocytochemistry was performed on 7–12 day old cultured (DIC) hippocampal neurons. Before labelling, neurons were washed with HEPES buffered saline containing glycine (0.01 mM) and treated with G1 for 30 min at room temperature (21–23 °C). For antagonist studies, neurons were pre-treated with pharmacological inhibitors for 30 min before G1 application. To label surface GluA1, neurons were incubated with an antibody against the N-terminal region of GluA1 (sheep anti-GluA1; 1:100; [47] at 4 °C, then fixed with 4% paraformaldehyde for 5 min. Surface GluA1 immunostaining was visualized by addition of an appropriate fluorescently conjugated anti-sheep secondary antibody (1:250, Life Technologies, UK) for 30 min. In a subset of experiments, neurons were permeabilized with 0.1% Triton X-100 (5 min) after GluA1 immunolabelling and fixed with 4% paraformaldehyde. A second primary antibody was applied to compare GluA1 surface immunostaining relative to PSD-95 (mouse anti-PSD-95; 1:500; Thermo Fisher, UK). An Alexa 568-conjugated anti-mouse secondary antibody (1:200; Thermo Fisher, UK) was then used to visualize PSD-95 labelling [46]. No labelling was observed after incubation with secondary antibodies alone.

### Analysis

A Zeiss LSM510 confocal imaging system was used for image acquisition, and 488 nm and 543 nm laser lines were used to excite the Alexa 488 and 568 fluorophores, respectively. Images were obtained in a single-tracking mode or multi-tracking mode for dual labelling experiments using a 15 s scan speed, whereas intensity of immunostaining was determined offline using LaserSharp software (Zeiss). Analysis lines (50 μm) were drawn along randomly selected dendritic regions and mean intensity of GluA1 surface staining was calculated for each selected dendrite [43, 46]. For synaptic co-localization experiments, surface GluA1 immunolabelling was compared with dendritic PSD-95 immunostaining, and number of GluA1-positive sites that co-localized with PSD-95-positive sites were counted and expressed as a percentage of PSD-95-positive sites. Data were obtained from at least 3 dendrites from a minimum of 4 randomly selected neurons for each treatment, and all data were obtained from at least 3 different cultures from different animals. Within a given experiment, all conditions, including illumination intensity and photomultiplier gains, were kept constant. To quantify experimental data obtained from separate days, data were normalized relative to mean fluorescence intensity in control neurons. For antagonist experiments, neurons treated with G1 in the presence of a pharmacological inhibitor were normalized to data from neurons treated with the inhibitor alone. All data are expressed as means ± SEM, and statistical analyses were performed using one-way analysis of variance for comparisons between multiple groups. *p* < 0.05 was considered significant with *n* representing the number of labelled dendrites analysed across experiments.

### Drugs

All drugs were dissolved in either aCSF or HBS and applied at the desired final concentration. Drugs used were dopamine (Sigma Aldrich, UK), G1 (Tocris, UK), G15 (Tocris UK), G36 (Tocris, UK), 17β‐estradiol (E2, Sigma Aldrich, UK), D-AP5 (HelloBio, UK), wortmannin (HelloBio, UK), LY294002 (HelloBio, UK), PD98059 (HelloBio, UK), U0126 (HelloBio, UK), philanthotoxin (Sigma Aldrich, UK), NVP-AAM007 (Sigma Aldrich, UK), ifenprodil (Tocris, UK), picrotoxin (Tocris, UK) and CGP55845 (Tocris, UK).

## Results

### E2-induced cLTP is blocked by a GPER1 antagonist

**Fig. 1:**
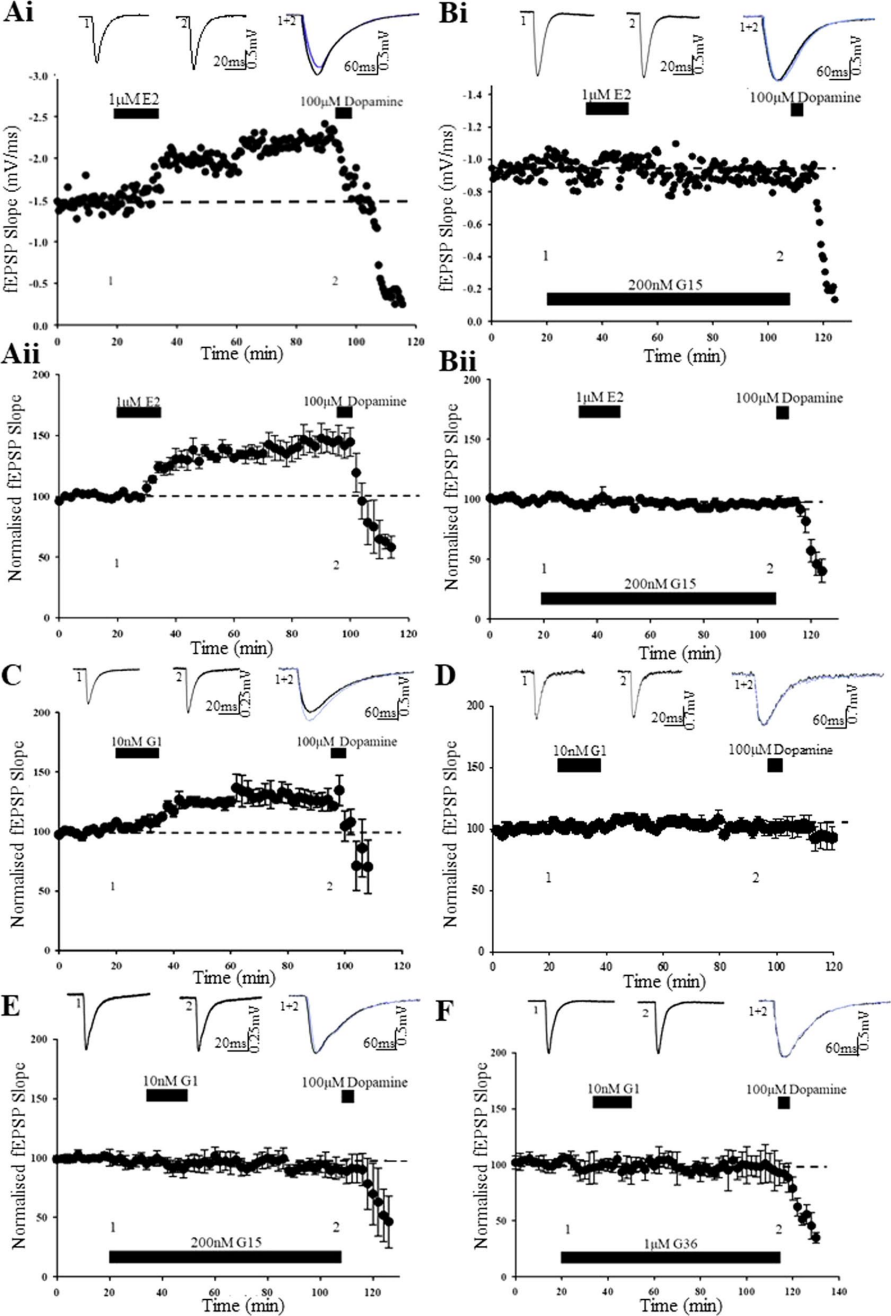
GPER1 activation results in a persistent increase in excitatory synaptic transmission. **Ai**, **Bi**. Representative experiment showing effects of estrogen (1 µM; 15 min) on excitatory synaptic transmission at TA-CA1 synapses in juvenile male hippocampal slices. Application of E2 resulted in a persistent increase in excitatory synaptic transmission (**Ai**) that was blocked by the GPER1 antagonist, G15 (**Bi**). At the end of experiments, addition of dopamine (100 µM; 5 min) inhibits synaptic transmission, confirming stimulation of TA input. **Aii**–**Bii**, Plots of pooled data demonstrating that E2 increases synaptic efficacy (**Aii**) and this effect is blocked by G15 (**Bii**). In this and subsequent figures, each point is the average of four successive responses and representative fEPSPs are shown above each plot and for the time indicated. **C**, **D** Plots of pooled data demonstrating that addition of the GPER1 agonist, G1 (10 nM; 15 min) resulted in a persistent increase in synaptic transmission at TA-CA1 synapses (**C**) but was without effect at SC-CA1 synapses (**D**). Note the lack of effect of dopamine at SC-CA1 synapses. **E**, **F** Pooled data showing that the effects of G1 are due to activation of GPER1 as G1 effects were blocked by the selective GPER1 antagonists, G15 (200 nM; **E**) and G36 (1 µM; **F**)

We have shown previously that acute exposure to E2 (1 µM; 15 min) induces a novel form of cLTP at juvenile TA-CA1 synapses [19]. As there is growing evidence that E2 is an agonist at GPER1, we assessed the role of GPER1 in E2-mediated cLTP at this synapse, using the GPER1 selective antagonist, G15 [21]. Application of G15 (200 nM, 60 min) had no effect on basal excitatory synaptic transmission (101 ± 2.9% of baseline; *n* = *4; F[1,3]* = *0.188; p* > *0.05*). In agreement with previous work [19], addition of E2 (1 µM) for 15 min resulted in a rapid potentiation of synaptic transmission (to 145 ± 9.5% of baseline, *n* = *4; F[1,3]* = *22.399; p* < *0.01*; Fig. 1A). In a subset of G15-treated slices (*n* = *5 out of 8 slices*), application of E2 (1 µM; 15 min) failed to evoke a significant increase in excitatory synaptic transmission (96 ± 1.7% of baseline; *n* = *5; F[1,4]* = *1.909; p* > *0.05;* Fig. 1B), suggesting possible involvement of GPER1 in mediating the actions of E2 at juvenile TA-CA1 synapses.

### The GPER1 agonist, G1 induces LTP at TA-CA1 synapses

Previous work has identified that E2 modulates excitatory synaptic transmission at SC-CA1 synapses via activation of GPER1 [48], and our data suggests that activation of GPER1 is involved in cLTP induced by E2 at TA-CA1 synapses. G1 has been shown to selectively bind to GPER1 at low nM concentrations, without significantly interacting with ERα or ERβ [12, 21]. Therefore, to assess the effects of GPER1 activation on excitatory synaptic transmission at juvenile TA-CA1 synapses, various concentrations (range 1–10 nM) of the GPER1 agonist, G1 were examined. Addition of 1 nM G1 (15 min) failed to significantly alter basal synaptic transmission (96 ± 4.7% of baseline; *n* = *5; F[1,4]* = *0.758; p* > *0.05;* not shown*).* However, application of 10 nM G1 (15 min) induced a persistent increase in excitatory synaptic transmission (to 129 ± 6.4% of baseline; *n* = *5; F[1,4]* = *11.432; p* < *0.01;* Fig. 1C). These data indicate that at higher concentrations, G1 induces a novel form of cLTP at juvenile TA-CA1 synapses.

As E2 also potently influences synaptic transmission at SC-CA1 synapses via activation of GPER1 [38], in parallel studies we compared the effects of G1 at juvenile male SC-CA1 synapses. In contrast to the actions at TA-CA1 synapses, addition of 10 nM or 1 µM G1 (15 min), had no effect on basal excitatory synaptic transmission at SC-CA1 synapses, as the magnitude of synaptic transmission was unaltered in the presence of either 10 nM G1 (102 ± 2.1% of baseline, *n* = *5; F[1,4]* = *0.136; p* > *0.05*) or 1 µM G1 (101 ± 4.3% of baseline; *n* = *5; F[1,4]* = *0.165; p* > *0.05*; Fig. 1D). These data suggest that the ability of G1 to influence excitatory synaptic transmission is restricted to TA-CA1 synapses, in juvenile male hippocampus.

To further verify involvement of GPER1 at TA-CA1 synapses, the effects of two GPER1-selective antagonists, G15 and G36, were assessed. Application of either G15 (200 nM; 60 min) or G36 (1 µM; 60 min) had no effect on basal synaptic transmission (101 ± 2.9% of baseline; *n* = *4; F[1,3]* = *0.188; p* > *0.05,* and 94 ± 2.4% of baseline; *n* = *4; F[1,3]* = *0.661; p* > *0.05*; respectively). In control slices, addition of G1 (10 nM; 15 min) caused a significant increase in synaptic transmission (to 132 ± 7.4% of baseline; *n* = *5; F[1,4]* = *13.484; p* < *0.001*). However, in slices treated with G15, addition of G1 (10 nM; 15 min) failed to alter synaptic transmission (98 ± 3.9% baseline; *n* = *5; F[1,4]* = *0.166; p* > *0.05;* Fig. 1E). Similarly, in G36-treated slices, addition of G1 (10 nM; 15 min) failed to alter the magnitude of excitatory synaptic transmission (98 ± 3.6% of baseline; *n* = *4; F[1,3]* = *0.279; p* > *0.05;* Fig. 1F). Together, these data indicate that the ability of G1 to induce cLTP at juvenile TA-CA1 synapses, requires activation of GPER1.

### GPER1-induced LTP has a postsynaptic expression mechanism

**Fig. 2:**
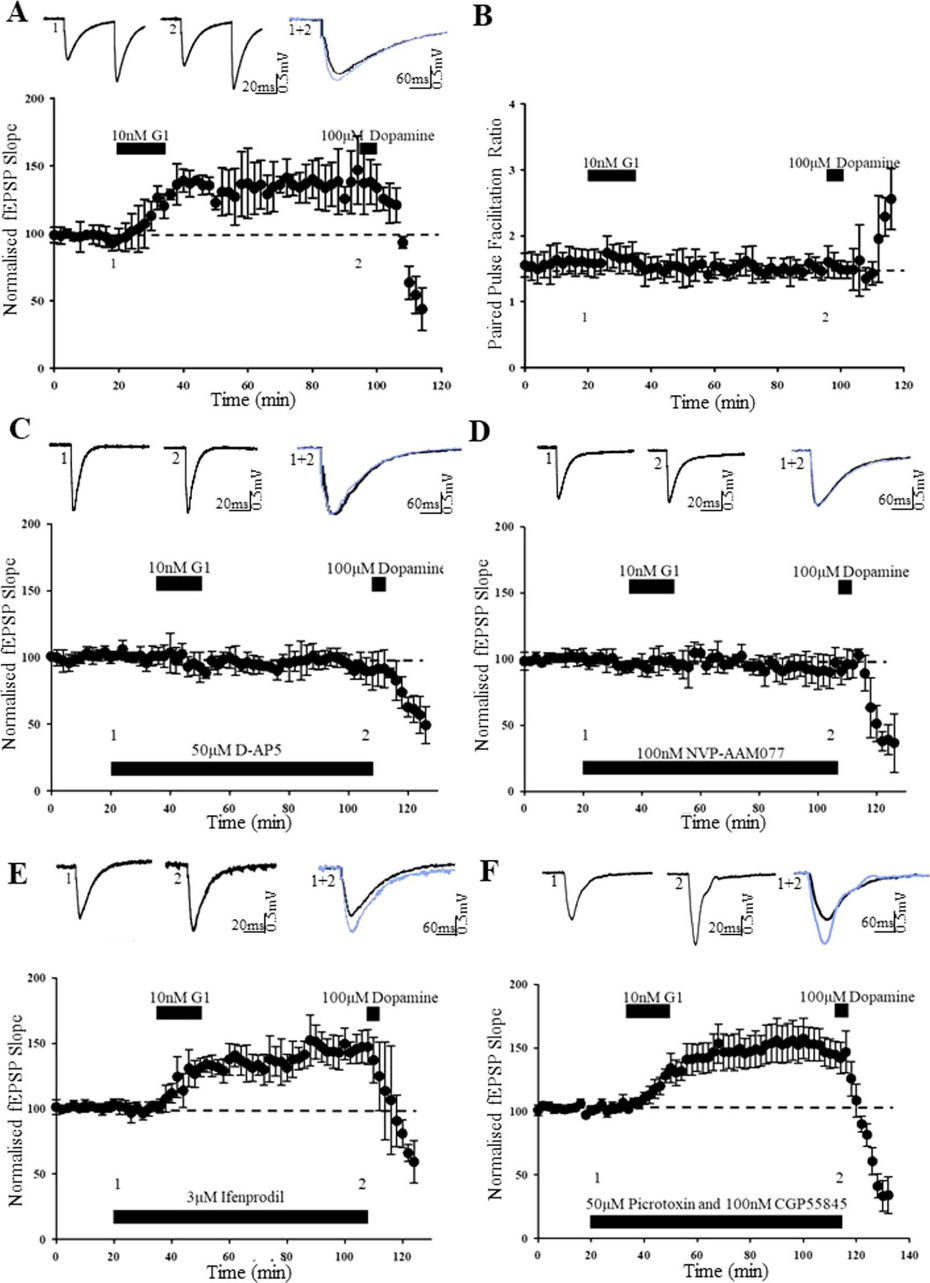
G1-induced cLTP involves a postsynaptic expression mechanism, and is dependent on NMDA, but not GABA, receptors. **A** Plot of pooled data illustrating the effects of 10 nM G1 (15 min) on synaptic transmission at TA-CA1 synapses. **B** Corresponding plot of the pooled paired pulse ratio (PPR) against time for the experiments shown in **A**. The effects of G1 were not associated with any change in PPR, indicating a postsynaptic expression mechanism. **C**–**F** Plots of pooled data illustrating the effects of G1 (10 nM; 15 min) on synaptic transmission in juvenile hippocampal slices. Activation of NMDA receptors was involved as G1 failed to increase synaptic efficacy in the presence of the NMDA receptor antagonist, D-AP5 (50 µM; **C**). **D**, **E** The ability of G1 to induce cLTP was blocked by inhibition of GluN2A subunits with NVP-AAM0077 (**D**), but was not altered by the GluN2B antagonist, ifenprodil (**E**). **F** G1-induced cLTP was independent of GABAergic inhibition as the effects of G1 were unaffected in the presence of GABA_A_ and GABA_B_ receptor antagonists, picrotoxin and CGP55845, respectively

In hippocampal neurons, GPER1 is expressed postsynaptically and it interacts with postsynaptic proteins, PSD-95 and SAP97 [1, 67]. GPER1 is also reported to modulate excitatory synaptic transmission at SC-CA1 synapses via a postsynaptic mechanism [48]. However, as GPER1 is also expressed at presynaptic sites [5], it is feasible that GPER1 located at either pre- or postsynaptic sites contributes to LTP induced at TA-CA1 synapses. Thus, to examine the locus of G1-induced cLTP, the effects on paired-pulse facilitation ratio (PPR were assessed, as changes in PPR likely indicate presynaptic alterations in neurotransmitter release probability. Application of G1 (10 nM, 15 min induced a significant increase in excitatory synaptic transmission (139 ± 9.2% of baseline; *n* = *4; F[1,3]* = *9.893; p* < *0.01;* Fig. 2A); an effect not accompanied by any significant change in PPR (from 1.60 ± 0.1% to 1.66 ± 0.1% of baseline; *n* = *4; F[1,3]* = *0.012; p* > *0.05;* Fig. 2B), suggesting likely involvement of a postsynaptic mechanism. At TA-CA1 synapses, dopamine depresses synaptic transmission via a presynaptic mechanism [49]. In accordance with this, application of dopamine not only depressed synaptic transmission, but this effect was accompanied by significant changes in PPR (from 1.6 ± 0.1% to 2.5 ± 0.2%, *n* = *4; F[1,3]* = *19.600; p* < *0.001;* Fig. 2B). These data indicate that GPER1-induced cLTP at TA-CA1 synapses is likely to involve a postsynaptic expression mechanism.

### Activation of GluN2A-containing NMDA receptors is required for GPER1-induced LTP

Synaptic activation of NMDA receptors is required for activity-dependent LTP at many central synapses and for E2-induced LTP at SC-CA1 synapses [59, 60]. Similarly, at adult TA-CA1 synapses, E2-induced LTP involves NMDA receptors [61] whereas ERα induces a novel form of NMDA-dependent form of LTP at juvenile TA-CA1 synapses [19]. Therefore, to assess the potential involvement of NMDA receptors, the effects of the competitive NMDA receptor antagonist, D-AP5 were evaluated. Treatment of slices with D-AP5 (50 µM, 60 min) had no significant effect on basal synaptic transmission (102 ± 0.9% of baseline; *n* = *5; F[1,4]* = *1.217; p* > *0.05*). In control slices, application of G1 (10 nM; 15 min) induced robust cLTP, as the magnitude of excitatory synaptic transmission was significantly increased (to 144 ± 5.4% of baseline; *n* = *5; F[1,4]* = *25.725; p* < *0.001*). In contrast, with D-AP5-treated slices, no significant change in excitatory synaptic transmission was detected following addition of G1 (95 ± 3.3% of baseline; *n* = *5; F[1,4]* = *0.017; p* > *0.05;* Fig. 2C). These data indicate that NMDA receptor activation is required for GPER1-mediated cLTP at juvenile TA-CA1 synapses.

GluN2 subunits control the biophysical and pharmacological characteristics of NMDA receptors [50], and distinct GluN2 subunits are implicated in activity-dependent synaptic plasticity at different stages of development and at distinct synapses [9, 44]. Previous studies have identified a role for GluN2B in ERα-induced LTP at juvenile TA-CA1 synapses [19], and in E2-induced LTP at adult TA-CA1 synapses [61]. Thus, the role of distinct GluN2 subunits in GPER1-induced cLTP was examined using subunit-selective NMDA receptor antagonists. Application of the putative GluN2A antagonist, NVP-AAM077 (100 nM, 60 min) or GluN2B antagonist, ifenprodil (3 µM; 60 min) had no effect on basal synaptic transmission (NVP-AAM077: 99 ± 3.7% of baseline; *n* = *5; F[1,4]* = *0.386; p* > *0.05;* Ifenprodil*:* 99 ± 1.1% of baseline; *n* = *5; F[1,4]* = *0.137; p* > *0.05),* respectively. In control slices, G1 (10 nM; 15 min) induced robust cLTP as synaptic transmission was significantly increased (to 144 ± 8.4% of baseline; *n* = *5; F[1,4]* = *17.136; p* < *0.01*). However, in NVP-treated slices (100 nM; 90 min), application of G1 (10 nM; 15 min) failed to alter synaptic transmission (92 ± 5.0% of baseline; *n* = *4; F[1,3]* = *0.449; p* > *0.05;* Fig. 2D). In contrast, with slices treated with ifenprodil (3 µM; 90 min), a significant increase in excitatory synaptic transmission was observed after addition of G1 (to 144 ± 5.2% of baseline; *n* = *4; F[1,3]* = *36.040; p* < *0.001;* Fig. 2E). Collectively, these data indicate that GPER1-mediated cLTP at TA-CA1 synapses involves activation of GluN2A-containing NMDA receptors.

### GPER1-mediated LTP occurs independently of GABAergic inhibition

As GPER1 has been detected on hippocampal GABAergic interneurons [7] and stimulation of the TA input can activate GABAergic interneurons [22], it is feasible that GABAergic synaptic transmission plays a role. To address this possibility, the effects of blocking GABA_A_ and GABA_B_ receptors using selective antagonists, namely picrotoxin and CGP55845, were evaluated. Co-application of picrotoxin (50 µM; 60 min) and CGP55845 (100 nM; 60 min) had no effect on basal synaptic transmission (100 ± 3.3% of baseline, *n* = *5; F[1,4]* = *0.792; p* > *0.05).* In line with previous findings, addition of G1 (10 nM; 15 min) evoked a significant increase in excitatory synaptic transmission (to 139 ± 4.35% of baseline, *n* = *5; F[1,4]* = *62.099; p* < *0.001*). Similarly, in slices treated with both GABA receptor antagonists, G1 induced an increase in excitatory synaptic transmission (to 150 ± 13.6% of baseline, *n* = *5; F[1,4]* = *17.544; p* < *0.001;* Fig. 2F); an effect not significantly different to the magnitude of G1-induced cLTP in control slices (*n* = *5; F[1,4]* = *0.688; p* > *0.05)*. These data indicate that GPER1-induced cLTP at TA-CA1 synapses is likely to be independent of GABA receptors.

### G1-induced LTP involves the insertion of GluA2-lacking AMPA receptors

**Fig. 3:**
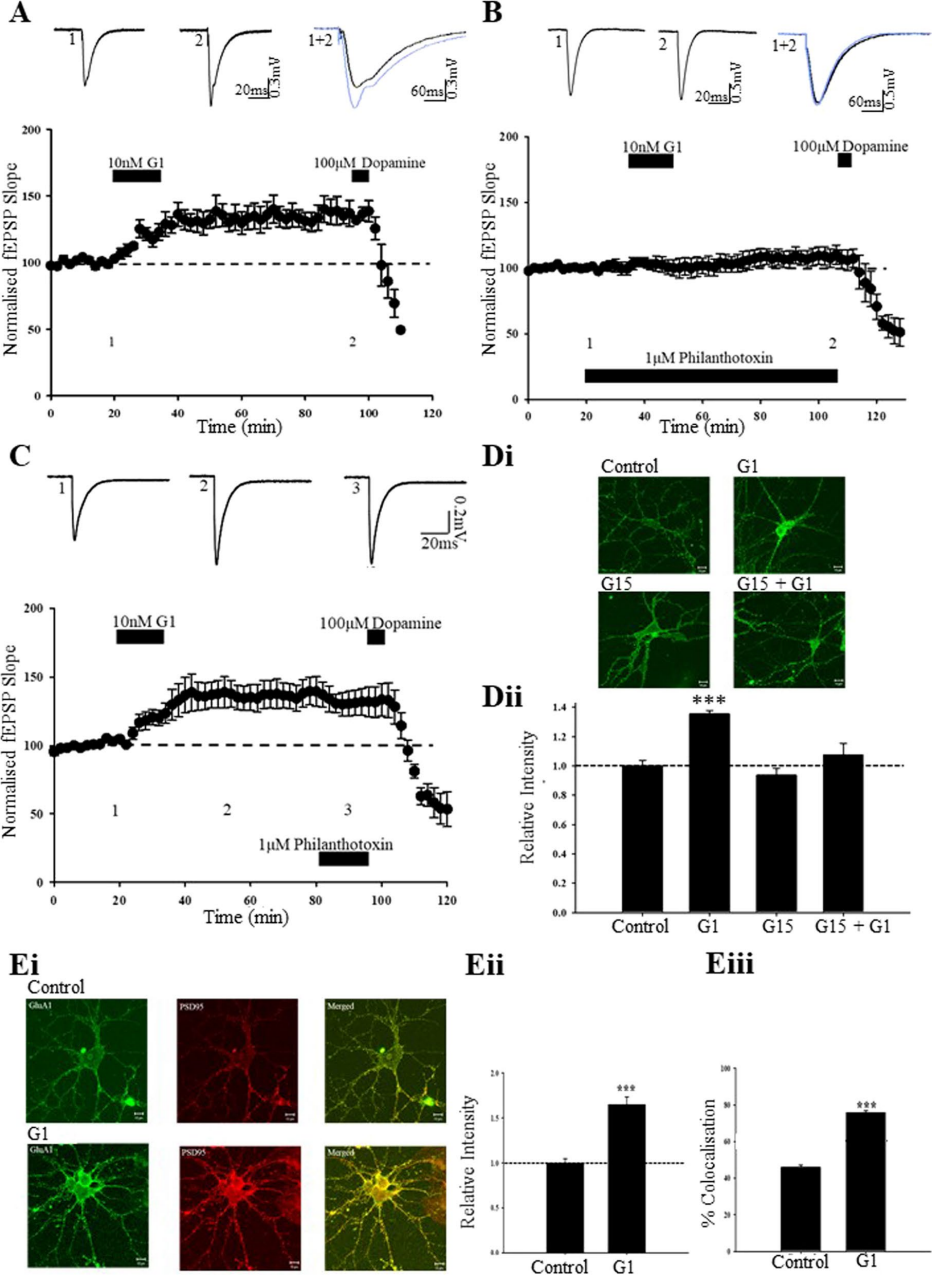
GPER1-induced cLTP involves synaptic insertion of GluA2-lacking AMPA receptors. **A**–**C** Plots of pooled data illustrating the effects on synaptic transmission at TA-CA1 synapses in juvenile hippocampal slices. **A** Application of G1 resulted in induction of cLTP, but prior exposure to philanthotoxin (Phtx; 1 µM; **B**) blocked G1-induced cLTP. **C** Application of philanthotoxin, 30 min after G1 failed to reverse the increase in synaptic efficacy induced by G1. **Di** Representative confocal images of surface GluA1 labelling in control hippocampal neurons (8–12 DIV) and after addition of G1, G15 and G1 plus G15. **Dii** Pooled data illustrating the relative effects on surface GluA1 immunolabelling in control neurons and after G1, G15, and G1 plus G15. G1 increases surface GluA1 expression via activation of GPER1. **Ei** Representative confocal images of surface GluA1 (green) and PSD-95 (red) labelling in control and G1 treated (DIV 8–13) neurons. G1 increases surface GluA1 that co-localises with PSD-95, indicating increased postsynaptic levels of GluA1. **Eii**, **iii** Pooled data indicating relative intensity of GluA1 (**Eii**) and % GluA1-PSD-95 co-localisation (**Eiii**) in control conditions and after G1 application. G1 increased GluA1 expression at hippocampal synapses

Activity dependent changes in excitatory synaptic strength involves trafficking of AMPA receptors to and from synapses [20], and synaptic insertion of GluA2-lacking AMPA receptors contributes to NMDA-dependent LTP evoked at juvenile SC-CA1 synapses [52]. Synaptic insertion of GluA2-lacking AMPA receptors is also implicated in activity-dependent LTP [41] and ERα-induced LTP [19] at TA-CA1 synapses. Therefore, the role of GluA2-lacking AMPA receptors in G1-induced cLTP was examined using philanthotoxin to inhibit GluA2-lacking AMPA receptors. In control slices, philanthotoxin (1 µM, 60 min had no effect on basal synaptic transmission (97 ± 1.2% of baseline *n* = *5; F[1,4]* = *0.556; p* > *0.05*). Addition of G1 alone (10 nM; 15 min) evoked a significant increase in excitatory synaptic transmission (to 138 ± 9.0% of baseline; *n* = *4; F[1,3]* = *16.772; p* < *0.001;* Fig. 3A). However, in slices pre-treated with philanthotoxin (1 µM; 90 min), G1 failed to induce cLTP (103 ± 6.0% of baseline; *n* = *5; F[1,4]* = *16.772; p* > *0.05;* Fig. 3B). In contrast, application of philanthotoxin, 20 min after the addition of G1, did not influence the magnitude of G1-induced cLTP (146 ± 8.8% of baseline; *n* = *6; F[1,5]* = *24.865; p* < *0.001*). Similarly, application of philanthotoxin, 60 min after G1, had no effect on the magnitude of G1-induced cLTP (131 ± 9.4% of baseline; *n* = *4; F[1,3]* = *7.816; p* < *0.01;* Fig. 3C). Together these data suggest that insertion of GluA2-lacking AMPA receptors is involved in the induction phase of GPER1-induced cLTP at TA-CA1 synapses.

### G1 increases synaptic insertion of GluA1-containing AMPA receptors

As our data suggest that GPER1 activation influences AMPA receptor trafficking to synapses, immunocytochemical techniques were performed to directly assay the cell surface density of AMPA receptors using an antibody against the AMPA receptor subunit, GluA1 on hippocampal neurons [47]. Exposure of hippocampal neurons to G1 (10 nM) for 15 min increased GluA1 surface immunolabelling (to 136 ± 2.2% of control, *n* = *36; F[1,71]* = *64.780; p* < *0.001;* Fig. 3D). To verify the involvement of GPER1, the effects of the GPER1 antagonist, G15 were evaluated. Treatment with G15 (200 nM; 15 min) alone, had no significant effect on GluA1 surface staining per se (94 ± 4.7% of control; *n* = *36; F[1,71]* = *1.048; p* > *0.05;* Fig. 3D). However, in G15-treated neurons, the effects of G1 were abolished as no significant change in GluA1 surface immunolabelling was detected (105 ± 2.9% of control; *n* = *36; F[1,72]* = *0.708; p* > *0.05;* Fig. 3D). These data indicate that the G1-induced increase in GluA1 surface expression requires GPER1 activation.

As AMPA receptor density at synapses is a key determinant of excitatory synaptic strength, we examined the effects of G1 on synaptic AMPA receptors by comparing the degree of co-localisation between surface GluA1 and the synaptic marker, PSD-95 [45]. Exposure of neurons to G1 (10 nM, 15 min), significantly increased GluA1 surface expression (to 165 ± 8.6% of control; *n* = *36; F[1,71]* = *43.358; p* < *0.001;* Fig. 3E); an effect accompanied by an increase in % co-localisation between surface GluA1 and PSD-95 from 46 ± 1.3% to 76 ± 1.3% (*n* = *36; F[1,71]* = *263.005; p* < *0.001;* Fig. 3E). Following G1 treatment, PSD-95 labelling was not significantly different to control neurons (104 ± 6.5% of control; *n* = *36; F[1,71]* = *0.263; p* > *0.05*), indicating that G1 does not alter synapse density per se. These data indicate that GPER1 activation increases the density of GluA1-containing AMPA receptors at hippocampal synapses.

### GPER1-driven AMPA receptor insertion requires NMDA receptor activation

**Fig. 4:**
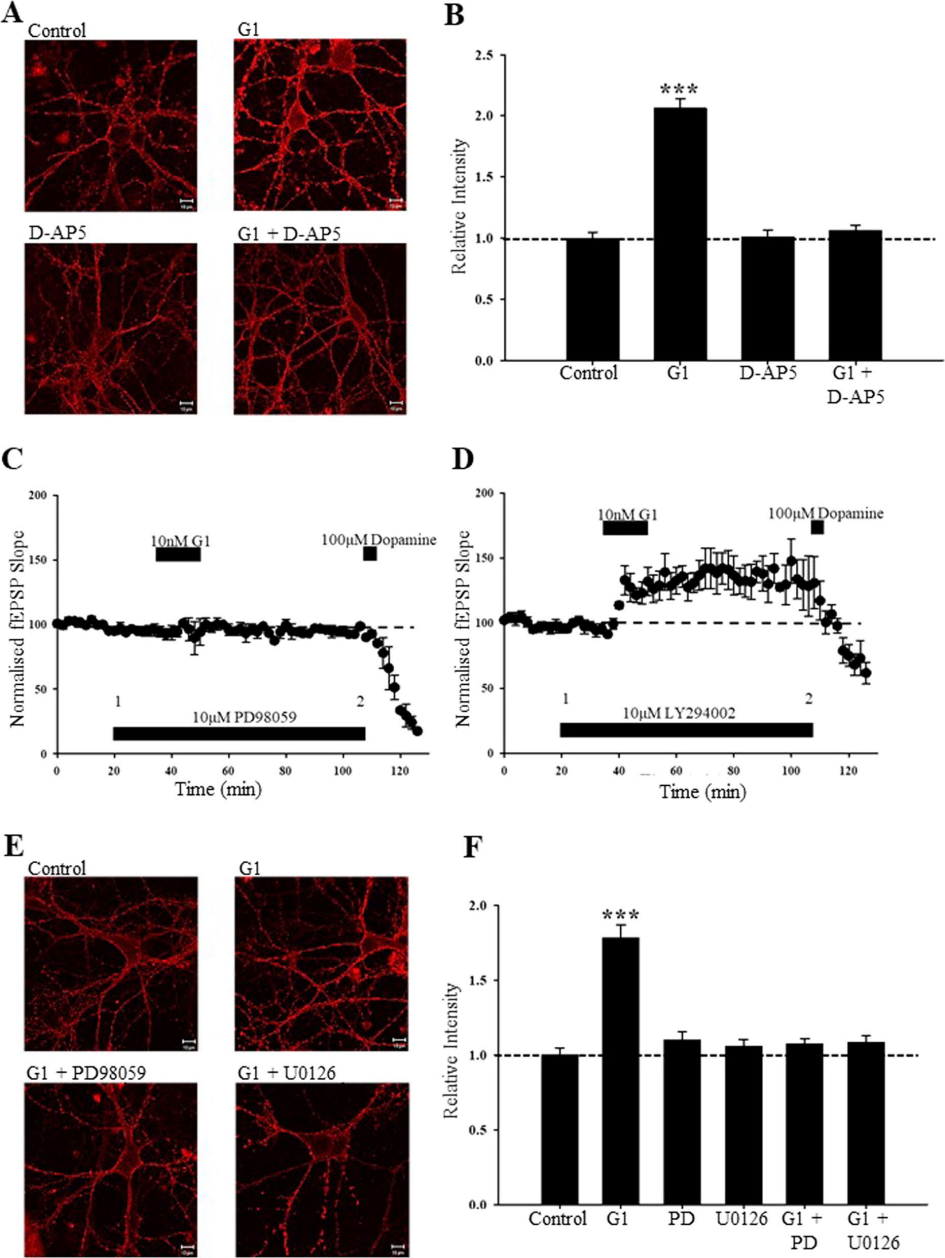
ERK signalling underlies GPER1-induced cLTP and synaptic insertion of GluA1. **A** Representative confocal images of surface GluA1 labelling in hippocampal neurons (DIV 8–12) in control conditions and after G1, D-AP5, and G1 plus D-AP5. NMDA receptor activation is required for G1 induced trafficking of GluA1-containing AMPA receptors. **B** Pooled data showing the relative effects on surface GluA1 labelling in control neurons and after exposure to G1, D-AP5 and G1 plus D-AP5. **C**, **D** Pooled data illustrating the effects on excitatory synaptic transmission in hippocampal slices. The ability of G1 to induce cLTP was blocked after ERK inhibition with PD98059 (**C**) but was unaffected in the presence of the PI3K inhibitor, LY294002 (**D**). **E** Representative confocal images of surface GluA1 labelling in hippocampal neurons (DIV 7–13) in control conditions, after G1, and in the combined presence of G1 and either PD98059 or U0126. **F** Pooled data showing the relative effects on surface GluA1 labelling in control neurons and after addition of G1, PD98059, U0126 or in the combined presence of G1 plus either PD98059 or LY294002. The G1-induced increase in GluA1 expression requires ERK signalling

As NMDA receptor activation can drive the movement of AMPA receptors to synapses [20], the role of NMDA receptors was explored using the competitive NMDA receptor antagonist, D-AP5. Treatment of neurons with G1 (10 nM, 15 min) significantly increased GluA1 surface immunolabelling (to 206 ± 7.6% of control; *n* = *36; F[1,71]* = *140.971; p* < *0.001;* Fig. 4A, B). Application of D-AP5 (50 μM; 15 min) alone had no effect on GluA1 surface staining (100 ± 5.4% of control; *n* = *36; F[1,71]* = *0.013; p* > *0.05*), however, in D-AP5-treated neurons, G1 (10 nM; 15 min) failed to significantly alter GluA1 surface expression (106 ± 4.5% of control; *n* = *36; F[1,71]* = *0.860; p* > *0.05;* Fig. 4A, B). These data indicate that NMDA receptor activation is required for GPER1-driven increase in AMPA receptor trafficking.

### GPER1-induced LTP involves ERK signalling but is independent of PI3K

As GPER1 can couple to a wide variety of signalling pathways, including ERK and PI 3K [23, 56], we investigated whether GPER1-induced cLTP at TA-CA1 synapses might involve one or more of these signalling pathways. To determine a potential role of ERK, the effects of two different inhibitors of MAPK activation, PD98059 and UO126, were investigated. Application of either PD98059 (10 µM, 60 min) or U0126 (10 µM; 60 min) had no effect on basal synaptic transmission (100 ± 1.0% of baseline; *n* = *5; F[1,4]* = *2.319; p* > *0.05;* and 99 ± 1.4% of baseline; *n* = *5; F[1,4]* = *0.143; p* > *0.05;* respectively). As before, application of G1 (10 nM; 15 min) readily induced LTP as synaptic transmission was significantly increased (to 147 ± 12.0% of baseline; *n* = *5; F[1,4]* = *12.688; p* < *0.001*). However, in slices treated with either PD98059 or U0126, G1 failed to significantly increase synaptic transmission (PD98059; 95 ± 4.0% of baseline; *n* = *4; F[1,3]* = *1.862; p* > *0.05;* Fig. 4C: U0126; 99 ± 1.4% of baseline; *n* = *4; F[1,3]* = *0.143; p* > *0.05*), suggesting involvement of ERK signalling in the effects of G1.

To explore the potential involvement of PI3K signalling, the effects of the PI3K inhibitor LY294002, were also examined. Application of LY294002 (10 µM; 60 min) had no significant effect on basal synaptic transmission (101 ± 4.8% of baseline; *n* = *5; F[1,4]* = *3.845; p* > *0.05*). Moreover, in interleaved slices treated with LY294002 (10 µM; 90 min), subsequent addition of G1 resulted in a persistent increase in synaptic transmission (to 133 ± 11.3% of baseline; *n* = *5; F[1,4]* = *33.041; p* < *0.01;* Fig. 4D). Together these data indicate that GPER1-induced cLTP involves stimulation of ERK, but not PI3K, signalling.

### ERK signalling is required for GPER1-driven insertion of AMPA receptors in hippocampal neurons

To verify if ERK signalling also underlies GPER1-driven AMPA receptor trafficking, the effects of two ERK inhibitors were examined. Treatment of hippocampal neurons with either PD98059 (10 µM; 15 min) or UO126 (10 µM; 15 min) had no effect on surface GluA1 staining per se (PD98059: 108 ± 5.3% of control; *n* = *36; F[1,71]* = *1.989; p* > *0.05;* U0126: 105 ± 4.39% of control; *n* = *36; F[1,71]* = *0.837; p* > *0.05;* Fig. 4F). In control neurons treated with G1 (10 nM; 15 min), GluA1 surface immunolabelling was significantly increased (to 178 ± 8.3% of control; *n* = *36; F[1,71]* = *67.275; p* < *0.001;* Fig. 4E, F). However, in PD98059-treated neurons, G1 failed to alter GluA1 immunolabelling (107 ± 3.3% of control; *n* = *36; F[1,71]* = *1.715; p* > *0.05;* Fig. 4E, F). Similarly, in neurons treated with UO126 the ability of G1 (10 nM; 15 min) to increase surface GluA1 immunolabelling was inhibited (108 ± 4.7% of control; *n* = *36; F[1,71]* = *1.522; p* > *0.05;* Fig. 4E, F). Together, these data indicate that ERK signalling mediates GPER1-driven changes in AMPA receptor trafficking.

### GPER1-induced LTP occludes HFS-induced LTP at TA-CA1 synapses

**Fig. 5:**
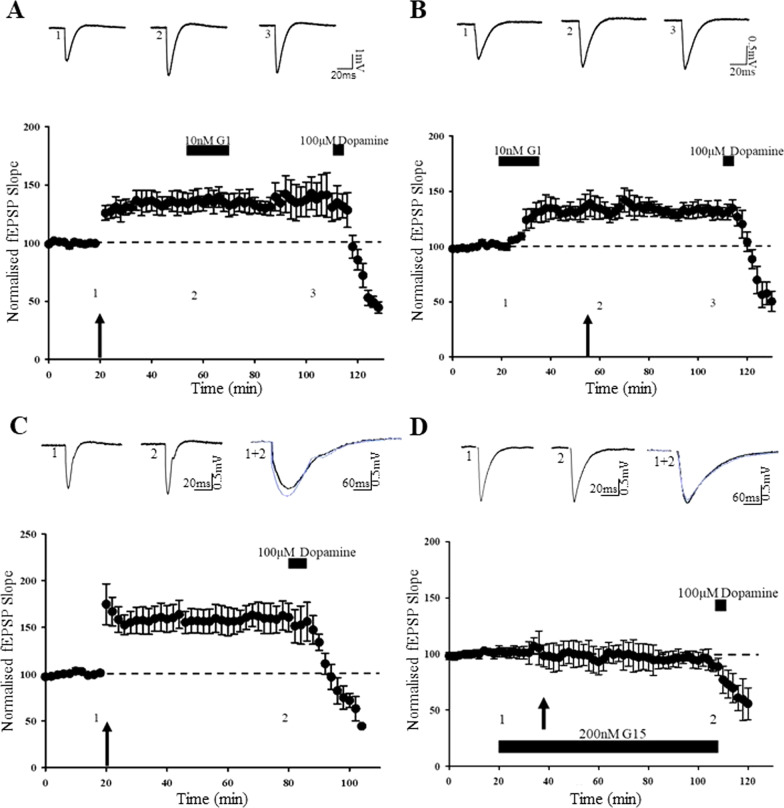
GPER1 is involved in activity-dependent LTP at TA-CA1 synapses. **A**–**D** Plots of pooled data illustrating effects on synaptic transmission at juvenile TA-CA1 synapses. **A** Delivery of HFS (shown by arrow) resulted in the induction of LTP, however subsequent addition of G1 failed to alter synaptic transmission. **B** Application of G1 induced cLTP, but subsequent delivery of HFS failed to increase synaptic transmission further. Activity-dependent LTP and GPER1-induced LTP share analogous expression mechanisms. **C** In control slices, HFS readily induced LTP. **D** In interleaved slices exposed to the GPER1 antagonist, G15 (200 nM), delivery of HFS failed to induce LTP. GPER1 activation is involved in activity-dependent LTP

Several studies have determined that activity-dependent LTP at TA-CA1 synapses is NMDA receptor dependent [2, 27, 54, 55] and requires ERK signalling and synaptic insertion of GluA2-lacking AMPA receptors [41]. Thus, analogous cellular mechanisms underlie activity-dependent LTP and GPER1-mediated cLTP at TA-CA1 synapses and consequently both processes may share similar expression mechanisms. To verify if overlapping expression mechanisms play a role, occlusion experiments were performed. In the first experiments, a HFS paradigm (100 Hz, 1 s) was applied to induce LTP and 30 min later slices were treated with G1 (10 nM; 15 min). Delivery of HFS readily increased the magnitude of synaptic transmission (to 135 ± 9.3% of baseline; *n* = *4; F[1,3]* = *18.003; p* < *0.001*), but subsequent application of G1 had no further effect on synaptic transmission (133 ± 8.1% of baseline; *n* = *4; F[1,3]* = *14.782; p* < *0.001;* Fig. 5A), indicating that HFS-induced LTP occludes G1-induced cLTP. In parallel studies, the reverse experiment was carried out, such that G1 (10 nM; 15 min) was initially applied to slices which significantly increased excitatory synaptic transmission (to 134 ± 9.1% of baseline; *n* = *5; F[1,4]* = *12.894; p* < *0.001;* Fig. 5B). Subsequent delivery of HFS failed to alter the magnitude of G1-induced cLTP (132 ± 6.8% of baseline; *n* = *5; F[1,4]* = *18.902; p* < *0.001;* Fig. 5B), indicating that GPER1-induced cLTP also occludes HFS-induced LTP at TA-CA1 synapses. These data demonstrate that HFS-induced LTP and GPER1-induced cLTP at TA-CA1 synapses occlude one another and are likely to have comparable expression mechanisms.

ER antagonists have been shown to significantly reduce or abolish hippocampal LTP [28, 30] and a specific role for ERα in activity-dependent LTP at both SC-CA1 and TA-CA1 synapses has been demonstrated [19, 66]. Given that our data indicates that GPER1 modulates synaptic efficacy at TA-CA1 synapses, it is feasible that GPER1 plays a role in activity-dependent TA-CA1 LTP. Thus, to assess this possibility, the effects of the GPER1-selective antagonist G15 on HFS-induced LTP were examined. Application of G15 alone (200 nM, 60 min) failed to alter basal synaptic transmission (*n* = *4; p* > *0.05*). In control slices, delivery of HFS paradigm (100 Hz; 1 s) resulted in LTP induction as excitatory synaptic transmission was significantly increased (to 160 ± 15.3% of baseline; *n* = *5; F[1,4]* = *15.548; p* < *0.001;* Fig. 5C). However, in the presence of G15 (200 nM; 80 min), HFS failed to significantly increase synaptic transmission above basal levels (95 ± 3.2% of baseline; *n* = *5; F[1,4]* = *1.572; p* > *0.05;* Fig. 5D). These data suggest that activation of GPER1 contributes to the induction of activity-dependent LTP at TA-CA1 synapses.

## Discussion

Numerous studies indicate that estrogens have pro-cognitive properties, as E2 regulates key cellular processes linked to hippocampal learning and memory. Indeed, the ability of E2 to modulate excitatory synaptic transmission at hippocampal SC-CA1 synapses is well documented. However, the functional effects of estrogens at the anatomically distinct TA-CA1 synapse is less clear. Here we provide the first compelling evidence that activation of the non-classical estrogen-sensitive receptor, GPER1 induces a novel form of NMDA receptor-dependent cLTP at juvenile male TA-CA1 synapses. GPER1-induced cLTP has a postsynaptic locus of expression that requires activation of GluN2A-containing NMDA receptors. Additionally, stimulation of the ERK signalling cascade and subsequent insertion of GluA2-lacking AMPA receptors into synapses is crucial for GPER1-induced cLTP. Moreover, the expression mechanisms underlying GPER1-induced cLTP are analogous to activity-dependent LTP as there is occlusion between these two processes, and HFS-induced LTP is blocked by the GPER1 antagonist, G15. Together these data indicate a pivotal role for GPER1 in regulating excitatory synaptic strength at hippocampal TA-CA1 synapses.

Here we show that low nM concentrations of the GPER1-selective agonist, G1 evoke a persistent increase in excitatory synaptic transmission at juvenile TA-CA1 synapses, which mirrors the effects of E2 at this synapse, at the same age [19]. The ability of G1 to induce cLTP was completely blocked by two distinct GPER1-selective antagonists, G15 and G36, thereby confirming a role for GPER1. Here, the modulatory effects of G1 were restricted to TA-CA1 synapses, as G1 failed to influence synaptic transmission at SC-CA1 synapses. The lack of sensitivity of SC-CA1 synapses to G1 is likely due to the stage of postnatal development, as G1-driven effects were observed in slices from immature hippocampus, whereas previous studies detected GPER1 effects in adult hippocampus [48]. However, the ability of GPER1 to influence hippocampal excitatory synaptic transmission parallels previous studies as G1 is also reported to significantly enhance synaptic transmission at adult SC-CA1 synapses in female rodents [48, 57]. In contrast, GPER1 activation has been linked to mGluR-dependent LTD at hippocampal mossy fibre synapses in male rats [15]. Consequently, the ability of GPER1 to bi-directionally modulate excitatory synaptic transmission may be sex-dependent as GPER1 is reported to have differential effects on synaptic transmission at SC-CA1 synapses that vary with sex [48].

A postsynaptic expression mechanism is implicated in activity-dependent LTP [41] and ERα-induced cLTP at TA-CA1 synapses [19]. Similarly, in this study GPER1-induced cLTP likely involves a postsynaptic mechanism as the effects of G1 on synaptic efficacy were not associated with changes in PPR. Similarly, at SC-CA1 synapses, G1-induced cLTP involves a postsynaptic mechanism as G1 increased mEPSC amplitude suggesting alterations in postsynaptic sensitivity to glutamate [48]. In support of a postsynaptic expression mechanism, interactions between GPER1 and postsynaptic PDZ proteins, like PSD-95 [1, 67] have been reported, with such interactions implicated in GPER1 signalling in dendritic spines [1].

Synaptic activation of NMDA receptors underlies activity-dependent synaptic plasticity at hippocampal SC-CA1 and TA-CA1 synapses [11, 41, 55]. Additionally, the ability of E2 to modify synaptic transmission and induce LTP at SC-CA1 and TA-CA1 synapses requires NMDA receptor activation [60, 61]. Here, we show that NMDA receptors are also pivotal for GPER1-induced cLTP at TA-CA1 synapses as the effects of G1 were blocked by D-AP5. In accordance with different GluN2 subunits being implicated in various forms of synaptic plasticity [57], GluN2A-containing NMDA receptors are crucial for GPER1-induced cLTP as selective antagonists of GluN2A, but not GluN2B, subunits blocked the effects of G1. This contrasts with the reported involvement of GluN2B-containing NMDA receptors in ERα-mediated LTP at juvenile TA-CA1 synapses [19], and suggests that molecularly distinct NMDA receptors contribute to the alterations in synaptic efficacy induced by activation of GPER1 and ERα.

Activation of NMDA receptors triggers movement of AMPA receptors to hippocampal synapses [20] and several studies support the notion that synaptic incorporation of GluA2-lacking AMPA receptors occurs during LTP induction at SC-CA1 and TA-CA1 synapses [41, 52]. Here we show that insertion of AMPA receptors into synapses is also pivotal for GPER1-induced cLTP, as prior application of the GluA2-lacking AMPA receptor inhibitor, philanthotoxin blocked the effects of G1 on synaptic efficacy. In contrast philanthotoxin failed to influence GPER-1-induced cLTP, when applied after G1 addition, suggesting that insertion of GluA2-lacking AMPA receptors is required for the induction but not maintenance phase of cLTP. These findings are consistent with the early phase of NMDA receptor dependent LTP involving delivery of GluA2-lacking AMPA receptors to CA1 synapses.

In this study, the surface expression of the AMPA receptor subunit GluA1 in cultured hippocampal neurons was significantly elevated by G1. This effect was apparent at sites labelled with PSD-95, suggesting that GPER1 triggers trafficking of GluA1 to synapses. The ability of GPER1 to regulate GluA1 trafficking is in line with recent evidence that G1 alters surface GluA1 expression in the hippocampal CA3 region [15], however, in contrast to this study, G1 attenuated, rather than increased GluA1 surface expression in CA3 neurons. The effects of GPER1 on AMPA receptor trafficking involve an NMDA receptor-dependent process, as blockade of NMDA receptors prevented the effects of G1. Together these data suggest that GPER1 activation is likely to trigger NMDA receptor-dependent insertion of AMPA receptors into hippocampal TA-CA1 synapses.

Although TA stimulation activates GABAergic interneurons [22] and hippocampal interneurons express GPER1 [6], GABAergic inhibition played no role as the ability of G1 to induce cLTP was unaffected in the combined presence of GABA_A_ and GABA_B_ receptor antagonists. Several different signalling pathways are activated by GPER1, including ERK and PI3K [23, 56, 66]. Here we show that the effects of GPER1 involve selective activation of ERK dependent signalling as pharmacological inhibition of ERK, but not PI3K, blocked the ability of G1 to induce cLTP in slices. The role of ERK in GPER1-induced cLTP displays parallels to other studies as GPER1 activation leads to an increase in ERK phosphorylation which underlies GPER1-mediated increased in excitatory synaptic transmission at SC-CA1 synapses [38]. ERK signalling is also implicated in E2-mediated synaptic actions at adult TA-CA1 synapses [61]. Previous studies indicate that activation of ERK signalling occurs downstream of NMDA receptors, and this signalling cascade is implicated in activity-dependent synaptic plasticity [63, 65]. The role of ERK also extends to AMPA trafficking events, as the ability of G1 to increase GluA1 surface expression was blocked by pharmacological inhibition of ERK, but not PI3K. In line with our data, ERK activity is required for synaptic insertion of hippocampal AMPA receptor subunits [53], whereas exocytosis of AMPA receptors following hippocampal LTP induction requires ERK signalling [51]. Moreover, activation of GluN2A-containing NMDA receptors promotes an increase in GluA1 surface expression that requires ERK signalling [33, 68]. Collectively, these data highlight a crucial role for ERK-dependent signalling in mediating GPER1-driven AMPA receptor trafficking to synapses and induction of cLTP.

**Fig. 6:**
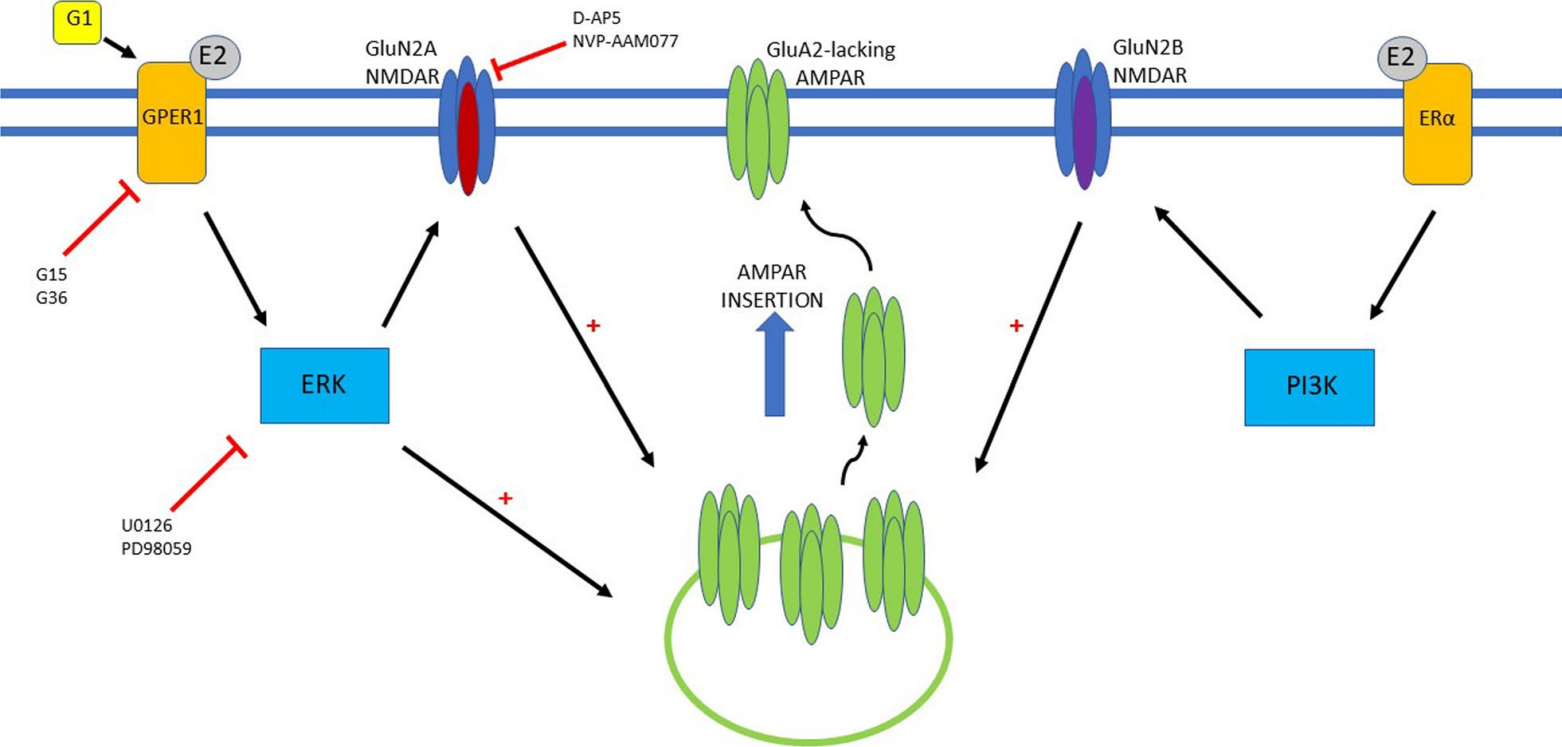
GPER1 activation results in induction of a novel form of NMDA receptor dependent LTP at juvenile TA-CA1 synapses. Schematic representation of the cellular events contributing to the induction of a novel form of LTP induced by activation of GPER1. Treatment with the selective GPER1 agonist, G1 or endogenous E2 results in the activation of GPER1 and stimulation of ERK-dependent signalling. This leads to activation of synaptic GluN2A-containing NMDA receptors and trafficking of GluA2-lacking AMPA receptors into hippocampal TA-CA1 synapses which in turn causes a persistent increase in synaptic efficacy. In parallel, E2 is also capable of activating ERα, which also promotes synaptic insertion of GluA2-lacking AMPA receptors and an increase in TA-CA1 synaptic efficacy, via a process involving PI3K-driven signalling and activation of GluN2B-containing NMDA receptors

Although we have identified that activation of NMDA receptors is required for GPER1-driven synaptic insertion of AMPA receptors and subsequent cLTP, the mechanisms underlying activation of NMDA receptors by GPER1 remain to be determined. Previous studies have identified that G1 can influence NMDA receptor function, by altering the phosphorylation status of NMDA receptors [40]. Consequently, it is feasible that activation of GPER1 stimulates ERK activation which in turn regulates NMDA receptor function by phosphorylating specific serine/threonine residues on NMDA receptors (Fig. 6).

It is well established that activity-dependent LTP is readily evoked at TA-CA1 synapses [19, 27, 41, 54]. Moreover, GPER1-induced cLTP displays similarities to activity-dependent LTP at TA-CA1 synapses, as both require NMDA receptors and involve insertion of GluA2-lacking AMPA receptors and ERK signalling [41]. In this study, HFS-induced LTP and GPER1-induced cLTP occlude one another as the magnitude of HFS-induced LTP was unaltered by subsequent application of G1. Similarly, the magnitude of cLTP induced by G1 was not affected after delivery of HFS, suggesting that both events have overlapping expression mechanisms.

Activity-dependent hippocampal LTP at SC-CA1 and TA-CA1 synapses is significantly reduced in the presence of ER antagonists [28, 30, 41] highlighting the importance of ERs for the induction of LTP. In accordance with this, HFS failed to induce TA-CA1 LTP in the presence of GPER1-selective antagonist, G15, suggesting that activation of GPER1 is required for the induction of activity-dependent LTP at this synapse. Our previous studies have also identified a role for ERα in the induction of LTP at juvenile TA-CA1 synapses [19]. But in contrast to the current study, completely divergent cellular mechanisms underlie ERα-induced LTP, with PI 3 kinase and GluN2B-containing NMDA receptors playing a key role [19]. In view of the fact that there are differences in the affinity of E2 for GPER1 and ERα [37, 56], it is likely that the relative contribution of GPER1 or ERα to the induction of activity-dependent LTP will depend not only on the brain concentrations of E2, but also the synaptic expression of the two oestrogen sensitive receptors.

It is widely accepted that TA-CA1 synapses contribute to hippocampal-dependent spatial memory, as impairments in spatial memory are evident in mice with lesions in the TA pathway [17], and reductions in TA-CA1 synapse excitability are reported to coincide with impaired spatial memory [32, 39]. Recent studies in humans indicate that TA-CA1 synapses have a crucial role to play in formation of episodic memories [62]. Consequently, as GPER1 activation results in persistent alterations in the strength of excitatory TA-CA1 synapses, it is likely that GPER1 also impacts episodic and spatial memory processes. Recent behavioural studies support this notion, as GPER1 activation is linked to enhanced spatial recognition memory [31, 42] and episodic memory [26, 34] whereas administration of the GPER1-selective antagonist G15 results in impairments in spatial memory [29].

Degeneration of the TA pathway is known to occur in Alzheimer’s disease (AD) and deficits in TA-CA1 synaptic plasticity is observed in the early stages of AD [13, 18]. As GPER1 activation has been shown to improve cognitive function in a mouse model of AD [36], the effects of GPER1 on TA-CA1 synaptic efficacy is also likely to have implications for memory and cognitive function in health and in age-related neurodegenerative conditions like AD.

## Data Availability

Data supporting the findings of this study are available from the corresponding author upon reasonable request.

## Abbreviations

AMPA: α-Amino-3-hydroxy-5-methyl-4-isoxazolepropionic acid
E2: 17β Estradiol
ER: Estrogen receptor
fEPSP: Field excitatory postsynaptic potential
GPER1: G-protein coupled estrogen receptor 1
HFS: High frequency stimulation
LTD: Long-term depression
LTP: Long-term potentiation
NMDA: *N*-Methyl-d-aspartate
PI3K: Phosphoinositide-3-kinase
PPR: Paired pulse facilitation ratio
SC: Shaffer-collateral
TA: Temporoammonic
